# Cigarette smoke stimulates the stemness of renal cancer stem cells via Sonic Hedgehog pathway

**DOI:** 10.1038/s41389-018-0029-7

**Published:** 2018-03-13

**Authors:** Weiwei Qian, Xiaochuan Kong, Tao Zhang, Dengdian Wang, Jin Song, Yuan Li, Xiaoting Li, Hao Geng, Jie Min, Qi Kong, Jie Liu, Zhiqi Liu, Daming Wang, Zhiqiang Zhang, Dexin Yu, Caiyun Zhong

**Affiliations:** 1 Department of Urology, The Second Affiliated Hospital of Anhui Medical University, Hefei, 230032 China; 2 Department of Nutrition and Food Safety, School of Public Health, Nanjing Medical University, Nanjing, 211166 China; 3 Department of Oncology, The First Affiliated Hospital of Anhui Medical University, Hefei, 230032 China

## Abstract

Cancer stem cells (CSCs) are essentially responsible for tumor initiation, growth, progression, metastasis and recurrence, and cigarette smoke (CS) is closely involved in the occurrence and development of kidney cancer. However, the effect of CS on renal CSCs has not been elucidated yet. In the present study, tumorsphere formation assay was used to enrich renal CSCs from 786-O and ACHN cells. We illustrated that CS effectively promoted renal CSCs stemness by enhancing tumorsphere formation, increasing the expression of renal CSCs markers (CD133, CD44, ALDHA1, Oct4, and Nanog) and elevating CD133^+^ cell population. Moreover, our results showed that CS triggered the activation of Sonic Hedgehog (SHH) pathway, while inhibition of SHH pathway dampened the promotive effects of CS on renal CSCs. Finally, higher levels of renal CSCs markers and SHH pathway-related proteins were observed in kidney cancer tissues from smokers than non-smoking cancer tissues. Taken together, these results demonstrated the important role of SHH pathway in regulating CS-induced renal CSCs stemness augment. Findings from this study could provide new insight into the molecular mechanisms of CS-elicited stemness of renal CSCs.

## Introduction

Among urologic tumors, renal cell carcinoma (RCC) is characterized as the highest cancer-specific mortality rate, and the 5-year survival rate for patients with metastatic disease is only 12%^1^. High metastatic index and resistance to radiation and chemotherapy of RCC are responsible for unpredictable presentation and poor clinical outcome^2^. Therefore, discovery of novel approaches for the treatment of RCC is urgent.

Cancer stem cells (CSCs), a small subpopulation of cancer cells, are critically implicated in tumor occurrence, growth, progression, metastasis, therapy resistance, relapse, and poor prognosis^3,4^. CSCs possess several distinct features including clonogenic ability, self-renewal, expression of stem cell markers, growth in non-adhesive spheroids and multipotency capacity^5–7^. CSCs have been identified and isolated from numerous solid malignancies including RCC^8–10^. Herein, a better understanding of the molecular mechanisms of CSCs is necessary to overcome the current treatment limitations.

Sonic Hedgehog (SHH) signaling pathway has emerged as a critical component of CSCs. Aberration activation of SHH pathway has been implicated in the initiation and progression of multiple cancer types^11^. The activation of SHH protein relies on its binding to its receptor Patched (PTCH), which initiates a downstream signaling cascade, ultimately regulating the target genes including CD133, CD44, and Nanog^12^. In the absence of SHH, PTCH suppresses the transmembrane protein Smoothened (Smo) activity, which then represses Smo to activate an intracellular signal transduction cascade through Gli transcription factors^13,14^. There are three Gli transcription factors: Gli1 functions as a transcription activator, Gli2 and Gli3 can act as either repressor or activator, in a context-dependent manner^15^.

A large amount of epidemiological studies have demonstrated that cigarette smoke (CS) is a major established risk factor of RCC^16^. CS exposure increases the proportion of cancer stem-like cells in lung cancer cells and head and neck cancer cells^17^. To date, however, the underlying molecular mechanisms of CS on kidney CSCs stemness remain to be elucidated.

Therefore, the present study was designed to investigate whether SHH pathway is involved in CS-promoted stemness of kidney CSCs. These novel findings may open new avenues in search of potential interventional target of CS-associated RCC.

## Results

### Enrichment of renal CSCs by serum-free medium (SFM) culture

**Fig. 1:**
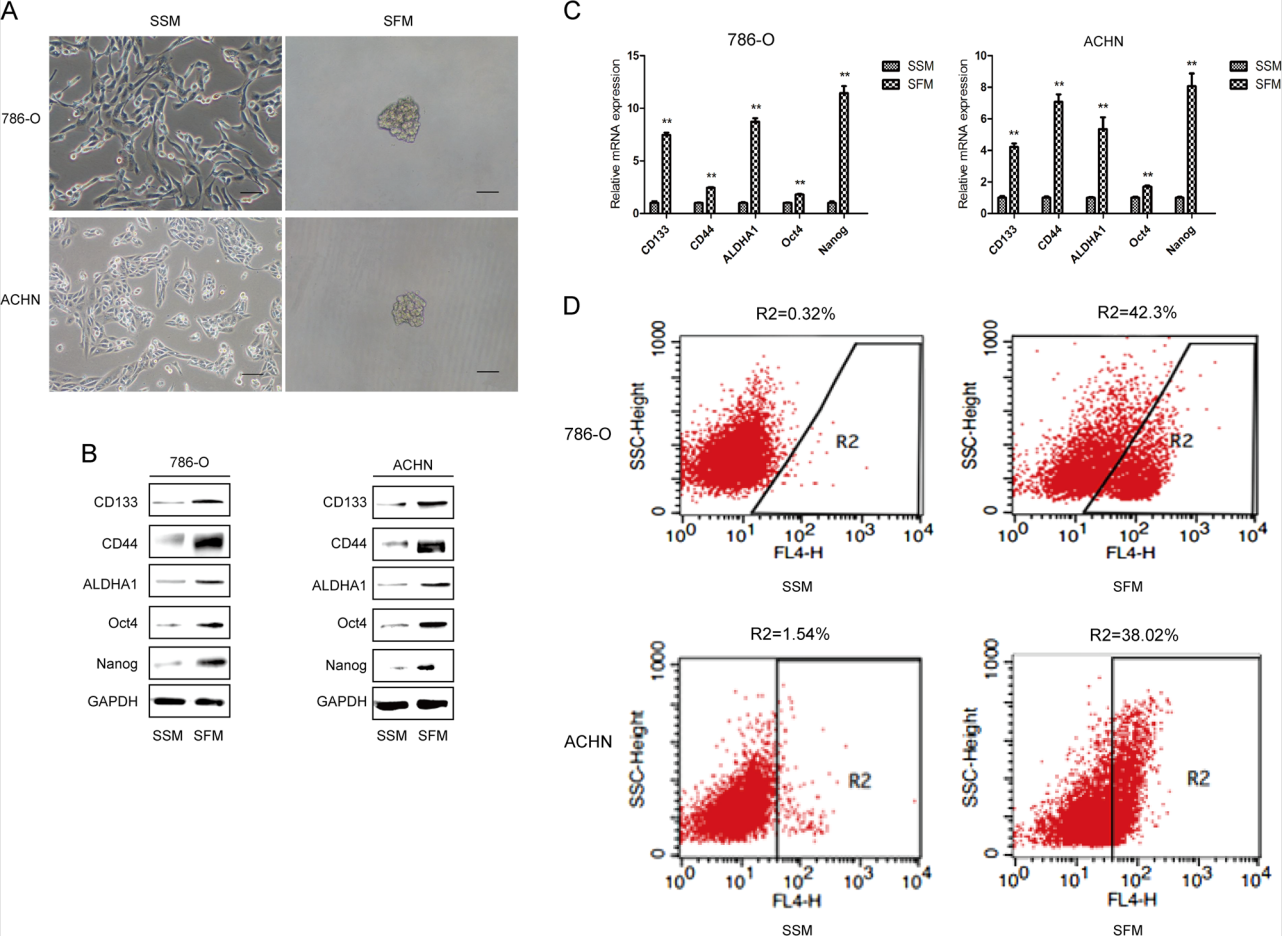
Tumorsphere formation assay of renal CSCs in SFM condition. 786-O and ACHN cells were grown in serum-supplied medium (SSM) and serum-free medium (SFM) for 5 days, respectively. **a** Representative images of tumorspheres. Bar, 100 μm. **b** The protein expression of renal CSCs markers (CD133, CD44, ALDHA1, Oct4, and Nanog) was measured by Western blotting. **c** The mRNA expression level of renal CSCs was measured by qRT-PCR. **d** The number of CD133^+^ cells in 786-O and ACHN cells was detected by flow cytometry. Data are expressed as mean ± standard deviation (SD). *p < 0.05, **p < 0.01 compared with SSM group

CSCs have the capacity to form three-dimensional structures or spheres, when cultured with SFM. Tumoresphere formation assay via SFM is widely used in isolation and enrichment of CSCs in vitro. To evaluate the characteristic of renal CSCs, we cultured two human RCC cell lines 786-O and ACHN under the conditions of SFM and serum-supplied medium (SSM), respectively. As shown in Fig. 1a, 786-O and ACHN cells grew adherently in SSM; under SFM, cells were able to form three-dimensional tumorspheres. Since renal CSCs express CSCs markers including CD133, CD44, ALDHA1, Oct4, and Nanog, their expression levels were determined in sphere-forming cells as well as in adherent cells. It was revealed that both protein and mRNA expression levels of the above indicated genes were markedly up-regulated in 786-O and ACHN tumorspheres cultured in SFM for 5 days (Figs. 1b, c). Moreover, flow cytometry analysis showed that higher percentage of CD133-positive cells was observed in those sphere-forming cells compared with adherent cells (Fig. 1d). Thus, these results suggested the characteristics of renal CSCs in 786-O and ACHN sphere-forming cells.

### CS promotes the stemness of renal CSCs

**Fig. 2:**
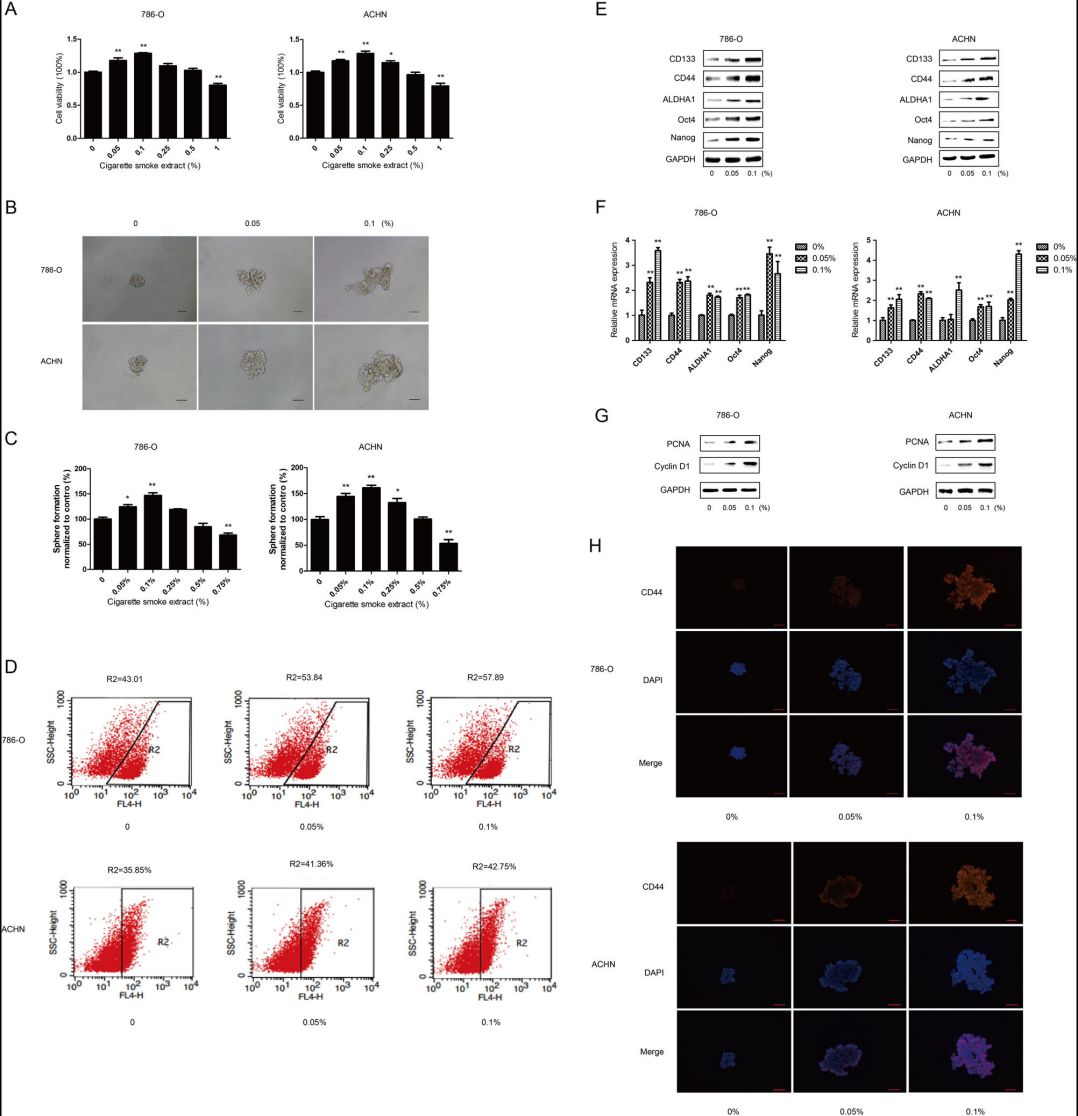
CSE promotes the characteristics of renal CSCs. Different concentrations of CSE were added to 786-O and ACHN tumorspheres for 5 days. **a** Cell viability was examined by CCK-8 assay. **b** The representative images of 786-O and ACHN sphere-forming cells were obtained. Bar, 100 μm. **c** The number of tumorspheres was counted and normalized to the control group. **d** Percentage of CD133^+^ cells after CSE treatment for 5 days. **e** Western blotting and **f** qRT-PCR were used to analyze protein and mRNA levels of renal CSCs markers. **g** Expression of cell proliferation associated protein was determined by western blotting. **h** Immunofluorescent staining of CD44 in 786-O and ACHN spheroids. Bar, 100 μm. Data are expressed as mean ± SD. *p < 0.05, **p < 0.01 compared with control

To determine the effect of CS extract (CSE) on the viability of the cells, 786-O and ACHN tumorspheres were treated with various concentrations of CSE for 5 days and cell viability was examined by CCK-8 assay. We found that a significant increase in cell viability was observed in 786-O cells at CSE concentrations of 0.05 and 0.1%, and in ACHN cells at CSE concentrations of 0.05, 0.1, and 0.25%; cell viability was significantly decreased at 1% CSE (Fig. 2a). Subsequently, the effect of CS on renal CSCs stemness was investigated. After treatment with CSE at 0.05 and 0.1%, CSE effectively promoted the size and numbers of 786-O and ACHN tumorspheres (Figs. 2b, c). Simultaneously, flow cytometry analysis showed that CSE increased the percentage of CD133-positive cells in those sphere-forming cells (Fig. 2d). Moreover, protein and mRNA expression levels of renal CSCs markers were significantly upregulated by CSE treatment (Figs. 2e, f). In addition, cell proliferation associated proteins (PCNA and Cyclin D1) were markedly increased by CSE (2 G). Immunofluorescent staining also revealed that CSE enhanced the expression of CD44 protein in 786-O and ACHN sphere-forming cells (Fig. 2h). Collectively, these data suggested that CSE promoted the stemness of renal CSCs.

### Upregulation of SHH pathway mediates the promotive effects of CS on renal CSCs

**Fig. 3:**
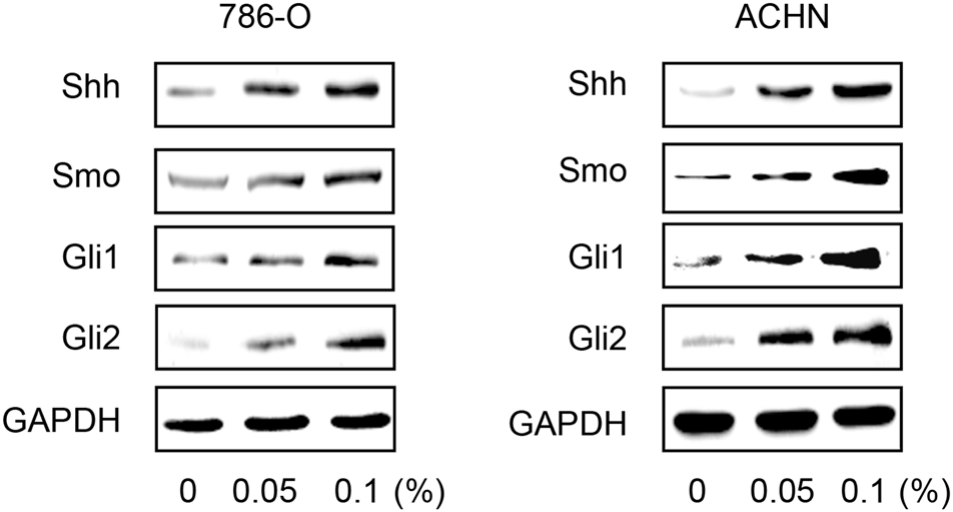
CSE activates SHH pathway in renal CSCs. Western blotting revealed the upregulation of SHH pathway after CSE treatment for 5 days

In order to determine whether SHH pathway was involved in CS-induced stemness of renal CSCs, we next examined the activation of SHH pathway in CSE-treated sphere-forming cells. As shown in Fig. 3, CSE significantly increased the expression of Shh, Smo, Gli1, and Gli2 in both 786-O and ACHN tumorspheres, indicating the activation of SHH pathway in renal CSCs.

**Fig. 4:**
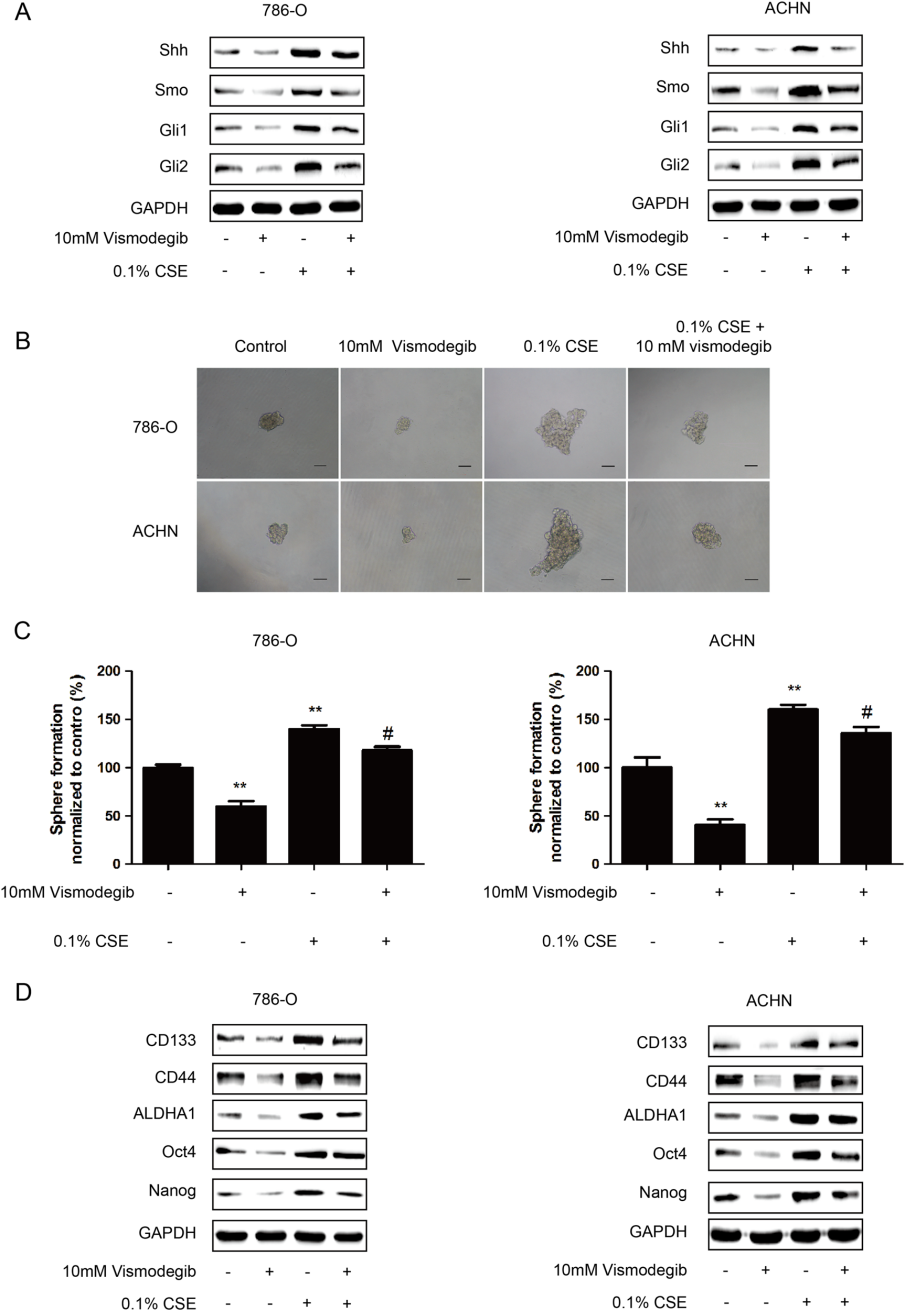
CSE promotes renal CSCs stemness via upregulating SHH pathway. **a**–**c** 786-O and ACHN tumorspheres were treated with 0.1% CSE with/without 10 mM Vismodegib for 5 days. **a** Western blotting analysis of SHH pathway proteins. **b** Images of tumorsphere formation. Bar, 100 μm. **c** The number of tumorspheres was calculated and normalized to control group. **d** Western blotting analysis of renal CSCs markers

To further examine the role of SHH pathway in the stimulative effect of CS on renal CSCs, Vismodegib, an inhibitor of Smo, was used to suppress SHH pathway. Our data showed that Vismodegib treatment inhibited the expression of SHH pathway-related proteins (Fig. 4a). It also showed that Vismodegib treatment resulted in lower tumorsphere formation (Figs. 4b, c) and reduced expression of renal CSCs markers in 786-O and ACHN sphere-forming cells (Fig. 4d). Meanwhile, we revealed that the stimulative effects of CS on SHH pathway, tumorsphere formation and renal CSCs markers were abrogated by Vismodegib treatment (Figs. 4a–d). Taken together, these data indicated that CS induced the stemness of renal CSCs through SHH pathway activation.

### Alteration of renal CSCs correlates with the smoking status of renal cell carcinoma patients

**Fig. 5:**
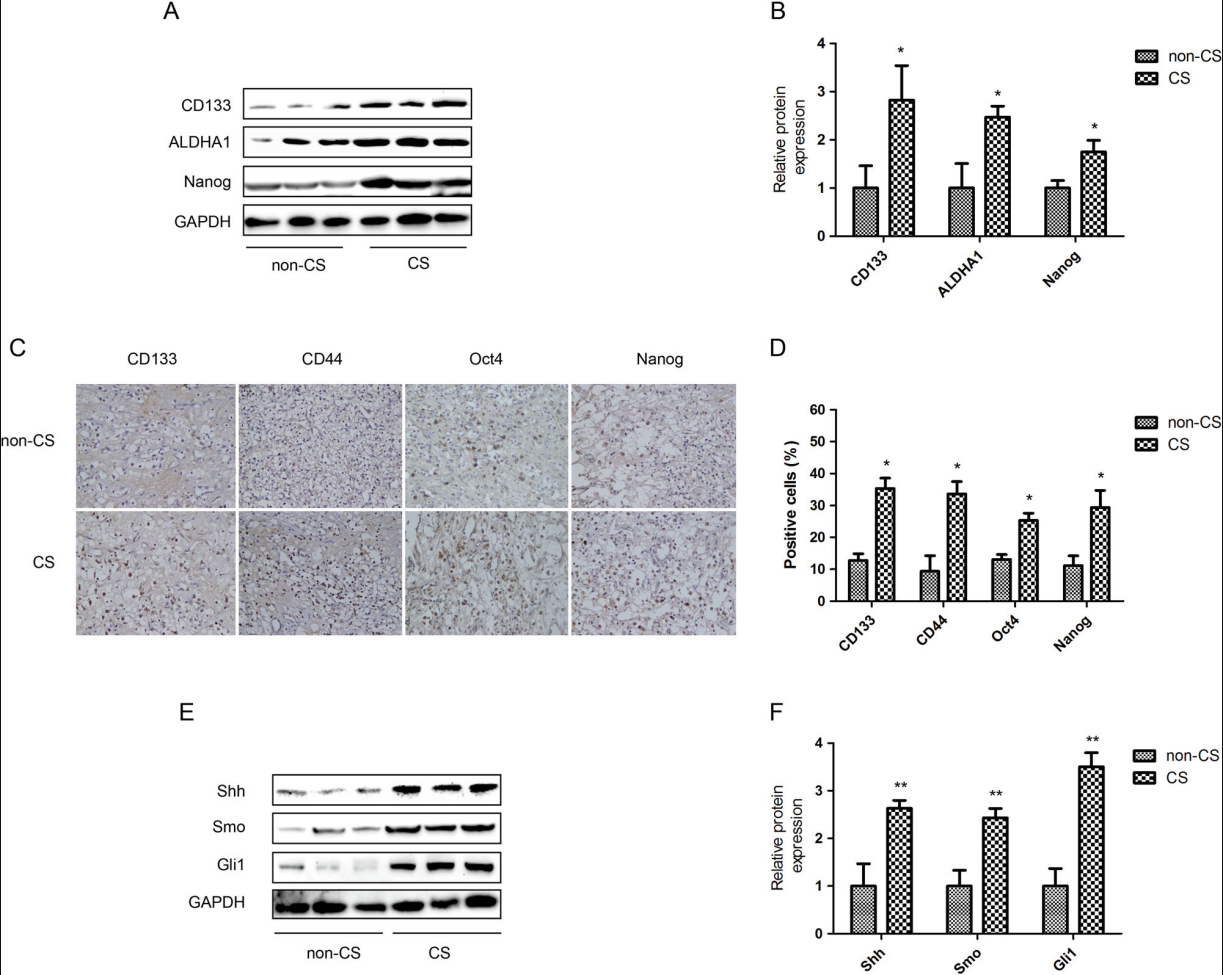
Alteration of renal CSCs correlates with the smoking status of renal cell carcinoma patients. Clinical kidney cancer specimens were collected from smoker and non-smoker renal cell carcinoma patients. **a** Western blotting of renal CSCs markers. **b** Quantitative analysis of Western blotting of renal CSCs markers. **c** Immunohistochemistry (IHC) assay of renal CSCs markers, 200× magnification. **d** Percentage of IHC positive cells in tissue samples. **e** Western blotting of SHH pathway proteins. **f** Quantitative analysis of Western blotting of SHH pathway proteins. non-CS: non-smoker cancer patients; CS: smoker cancer patients. Data are expressed as mean ± SD. *p < 0.05, **p < 0.01 compared with non-smoke cancer patients

The clinical characteristics of a total of 20 renal cell carcinoma patients were descripted in detail in Table 1. Postoperative tumor tissues were collected for western blotting and immunohistochemistry (IHC) staining to compare the expression of renal CSCs markers in smoker and non-smoker patients. Western blotting showed that higher expression levels of CD133, ALDHA1 and Nanog were observed in smoker tumor tissues than in non-smoker tumor tissues (Figs. 5a, b). In addition, these western blotting results of CSCs markers were further confirmed by IHC staining (Figs. 5c, d). Moreover, the expression levels of Shh, Smo and Gli1 were also notably increased in smoker tumor tissues (Figs. 5e, f).

**Table 1:** Clinical characteristics of patients (*n* = 20)

Parameters	Number of cases
*Gender*	
Male	12 (60%)
Female	8 (40%)
*Age*	
≦50	3 (15%)
>50–≦ 70	15 (75%)
>70	2 (10%)
*Tumor size (cm)*	
≦4	11 (55%)
>4–≦ 7	7 (35%)
>7	2 (10%)
*Tumor stage*	
pT1a	11 (55%)
pT1b	6 (30%)
pT2a	1 (5%)
pT2b	1 (5%)
pT3	1 (5%)
*TNM stage*	
I	17 (85%)
II	2 (10%)
III	1 (5%)
IV	0 (0%)

## Discussion

CSCs play a crucial role in the progress, metastasis and recurrence of human cancers. CS is a major risk factor of RCC. SHH pathway is critically implicated in tumorigenic process. However, the molecular mechanisms for the effects of CS on renal CSCs remain unclear. In the present study, we illustrated the pivotal role of SHH pathway in CS-induced enhancement of renal CSCs stemness. These findings could provide new insights into the molecular mechanism of kidney cancer occurrence and advancement.

It has been established that CSCs are able to form stable three-dimensional spheres in vitro. Hence, tumorsphere formation assay in SFM culture condition offers convenient approach for the enrichment and isolation of CSCs^18–20^. CSCs are characterized by the expression of distinct cell markers. CD133, CD44, ALDHA1, Oct4, and Nanog are widely used for identifying renal CSCs. CD133 has been used to isolate and identify a variety of CSCs including renal CSCs^21^. The expression of CD133 relates to the prognosis in renal cell carcinoma^22^. CD44 is also a clear renal CSC marker. There is a positive correlation between CD44 expression and tumor metastasis^23^. Besides, ALDHA1 positive cells present higher tumorigenicity than ALDHA1 negative counterparts^24^. Aberrant expression of Oct4 and Nanog, pluripotent stem cell markers of embryo and embryonic stem cells, participates in pluripotent initiation and differentiation^25^. In this study, we showed the tumorsphere formation capacity of both 786-O and ACHN cells cultured in SFM, and markedly increased expression levels of CD133, CD44, ALDH1A1, Oct4, and Nanog in sphere-forming cells. Meanwhile, flow cytometry analysis for the detection of CD133 positive cells was in line with the tumorsphere formation assay and Western blotting data. These results suggested the characteristics of CSCs in these renal cancer cells.

CS is widely considered as a significant and independent risk factor for RCC. In comparison with non-smokers, the risk for RCC has been increased about 50% in male and 20% in female smokers^26^. Besides, CS negatively impacts the overall and cancer-specific survival of renal cell carcinoma^27^. What is more, CS is closely associated with CSCs. CS induces the acquisition of CSCs properties in 40-passage transformed human bronchial epithelial cell^28^. However, no study has addressed the possible relationship between CS and renal CSCs so far. In the present study, we demonstrated that CS significantly enhanced renal CSCs stemness by increasing tumorsphere formation, upregulating the expression levels of renal CSCs markers (CD133, CD44, ALDH1A1, Oct4, and Nanog) and elevating the population of CD133 positive cells. Meanwhile, higher expression of renal CSCs markers was also revealed in RCC tumor tissues of smokers. Together, these data suggested that CS increased the stemness of renal CSCs.

Several embryonic signal pathways, such as SHH, Notch and Wnt/β-catenin pathways, have been implicated in CSCs. SHH signaling pathway is essential for the maintenance of CSCs^29^. In anaplastic thyroid cancer, SHH pathway maintains the self-renewal property of CSCs^30^. Inhibition of SHH pathway blocks pancreatic CSCs growth in vitro and in vivo^31^. In addition, it has been reported that SHH pathway closely correlates with kidney cancer. Activation of SHH pathway facilitates renal tumor growth^31^. By targeting SHH pathway, Vitamin D3 triggers its antitumor activity in renal cell carcinoma^32^. We showed in our study that CS induced the activation of SHH pathway in renal CSCs, as evidenced by increased expression levels of Shh, Smo, Gli1, and Gli2. Furthermore, we demonstrated that the promotive effects of CS on SHH pathway activation, tumorsphere formation, and renal CSCs markers expression were diminished by Vismodegib-elicited downregulation of SHH pathway. It is noteworthy that Vismodegib failed to totally rescue the effect of CS on renal CSCs, suggesting that CS-derived cancer stemness could also be driven by other signaling pathways. Liang et al reported that Wnt/β-catenin pathway modulates CS-triggered stemness in bladder tumor^33^. Besides, Naoya et al also revealed that CS-specific nitrosamine 4-(methylnitrosamino)-1-(3-pyridyl)-1-butanone increases lung cancer stem cell proportion through Wnt pathway^34^. These findings support our results. Finally, we showed that significantly increased expression levels of SHH pathway proteins as well as renal CSCs markers were observed in smoker tumor tissues than non-smoker tumor tissues. Taken together, these data revealed that the stimulative effect of CS on renal CSCs stemness was at least partially through the activation of SHH signaling pathway. In summary, the present study illustrated the important role of SHH pathway in CS-triggered enhancement of renal CSCs stemness. These findings may provide new insights into the molecular mechanism of CS-associated renal malignancy tumorigenesis and advancement.

## Materials and methods

### Cell culture and reagents

Human kidney cancer cell lines 786-O and ACHN were purchased from Chinese Academy of Typical Culture Collection Cell Bank (Shanghai, China), cultured in 37 °C, 5% CO_2_, and saturated humidity. 786-O cells were maintained in RPMI 1640 (Gibco, Carlsbad, CA, USA) medium supplemented with 10% fetal bovine serum (FBS) and 1% penicillin/streptomycin. ACHN cells grew in the same condition except in MEM (Gibco, Carlsbad, CA, USA) medium. Vismodegib was acquired from Medchem Express (NJ, USA). Epidermal growth factor (EGF) and basic fibroblast growth factor (bFGF) were acquired from Peprotech (Rocky Hill, NJ, USA). Insulin and 2% B27 were acquired from Gibco. Primary antibodies including CD133, CD44, ALDHA1, Oct4, Nanog, PCNA, CyclinD1, Shh, Smo, Gli1, Gli2, and GAPDH were purchased from Proteintech (Rocky Hill, NJ, USA). Anti-rabbit and anti-mouse second antibodies were purchased from ZSGB-BIO (Beijing, China). PCR primers of CD133, CD44, ALDHA1, Oct4, and Nanog were synthesized by Beijing Genomics Institute (Beijing, China).

### Tumorsphere formation assay

786-O and ACHN cells were seeded into 24-well culture plates at 5000 cells per well and cultivated in SFM [Dulbecco’s Modified Eagle’s Medium: Nutrient Mixture F-12 (DMEM/F12) (Gibco)] supplemented with 20 ng/mL EGF, 20 ng/mL bFGF, 5 μg/ml insulin and 2% B27, and half SFM with additions were renewed every other day. Tumorspheres were visualized by a microscope subsequently (Nikon, Japan).

To investigate the effect of CSE on renal CSCs stemness alterations, various concentrations of CSE were added to each well. After 5 consecutive days of treatment, the number and size of sphere-forming cells were obtained (only sphere diameter > 50μm was counted).

### Preparation of CS extract

CSE was daily prepared for each experiment immediately before use by combusting a filterless 3R4F reference cigarette (University of Kentucky, Kentucky, USA; each cigarette containing 9 mg tar and 0.8 mg nicotine) according to the reported method. After a cigarette was smoked by a vacuum, mainstream smoke was drawn through 10 ml pre-warmed (37 °C) FBS-free DMEM-F12 at the rate of 5 min/cigarette. Then the CSE stock solution was adjusted to pH 7.4 and then passed through a 0.22 μm-pore filter. The obtained mixture was defined as concentration of 100% CSE. Both 786-O and ACHN cells were treated with desired concentrations of CSE for 5 days. An unlit cigarette was performed with control solution following the same protocol.

### Western blotting analysis

Tumorspheres were collected, washed with ice-cold phosphated-buffer saline (PBS) and then solubilized in RIPA buffer (Thermo Scientific, USA) containing protease inhibitors. Protein concentration was measured by BCA Protein Assay Kit (Pierce, Rockforsd, WI, USA), and was then subjected to sodium dodecyl sulfate-polyacrylamide gel electrophoresis (SDS-PAGE) and transferred to polyvinylidene difluoride membranes (Millipore, Billerica, USA). After blocking with 5% defatted milk at room temperature, the transferred membranes were incubated with specific primary antibodies (1:500–1000 dilution) at 4 °C overnight and then followed by incubating with secondary antibodies. GAPDH was used as a loading control.

### Quantitative real-time polymerase chain reaction

Total RNA was extracted by TRIzol reagent (Invitrogen, Carlsbad, CA, USA) and RNA (1 μg) was reverse-transcribed into cDNA following the manufacturer’s instructions (abm, Canada).

The quantitative real-time PCR (qRT-PCR) was carried out by the Power SYBR Green Master Mix (Applied Biosystems, Foster City, CA, USA) and a LC96 real-time PCR detection system (Roche, Biosystems). Standardized by GAPDH, the mRNA expression levels of CD133, CD44, ALDH1A1, Oct4 and Nanog were measured. Fold changes of gene expression were obtained by the formula 2^−ΔΔCt^ using comparative threshold cycle (Ct). Primer sequences are listed below:

CD133-F, 5′-TACAACGCCAAACCACGACTGT-3′; CD133-R, 5′-TCTGAACCAATGGAATTCAAGACCCTTT-3′; CD44-F, 5′-GACACATATTGTTTCAATGCTTCAGC-3′; CD44-R, 5′-GATGCCAAGATGATCAGCCATTCTGGAAT-3′; ALDH1A1-F 5′-GCACGCCAGACTTACCTGTC-3′; ALDH1A1-R 5′-CCTCCTCAGTTGCAGGATTAAAG-3′; Oct4-F 5′-TGGGATATACACAGGCCGATG-3′; Oct4-R 5′-TCCTCCACCCACTTCTGAG-3′; Nanog-F 5′-TTTGTGGGCCTGAAGAAAACT-3′; Nanog-R 5′-AGGGCTGTCCTGAATAAGCAG-3′; GAPDH-F 5′-CAAGGTCACCATGACAACTTTG-3′;

GAPDH-R 5′-GTCCACCACCCTGTTGCTGTAG-3′

### Detection of CD133 positive cells by flow cytometry

786-O and ACHN adherent cells and tumorpheres were treated with selective concentrations of CSE for 5 days respectively, and cells were collected and washed by ice-cold PBS twice. Afterwards, 1 × 10^6^ cells were exposed to 1 μL APC-conjugated human monoclonal CD133/1(AC133) (Miltenyi Biotech, Teterow, Germany) antibody or isotype control antibody (Mouse IgG1) (Miltenyi Biotech) at 4 °C in the darkness for 10 min subsequently re-suspended in 400 μl PBS and detected by flow cytometry analysis.

### Cell proliferation assay

Cells grew in SFM at a density of 1 × 10^3^ cells/well in a 96-well plates were renewed half every other day. After 5 days, cell proliferation assay was conducted using cell counting kit 8 (CCK-8) according to the manufacturer’s instructions. After incubating at 37 °C for 3 h, cell viability was measured at 450 nm absorbance by a microplate reader (Titertek Instruments Inc, USA). Each experiment was performed in triplicates.

### Immunofluorescence staining

786-O and ACHN cells were seeded into 6-well culture plates at a density of 5 × 10^4^ per well. After 5 days, cells were fixed, washed, and then incubated with CD44 (1:200) antibodies at 4 °C overnight. Next day, cells were stained with Cy3-conjugated goat anti-rabbit secondary for 2 h. After washing three times, cells were incubated with DAPI for 15 min. Images were obtained by a reversed fluorescence microscopy (Nikon, Japan).

### Patients and tumor specimens

A total of 20 renal cell carcinoma patients were not treated with anti-tumor regimens before undergoing surgical operation in the Second Affiliated Hospital of Anhui Medical University, and their postoperational specimens were obtained. All the tissue sections were evaluated by experienced pathologists to confirm the diagnosis of renal cell carcinoma with WHO classification. The present study was approved by the Ethics Committee of Anhui Medical University. Written informed consent was obtained from the patients for this study.

### Immunohistochemistry (IHC)

IHC was used to analyze the expression of renal CSCs markers (CD133, CD44, Oct4, and Nanog) in samples of postoperative tumor tissues. The fixed specimens were paraffin-embedded and cut into 4 μm slides. Slides were then deparaffinized in xylene, hydrated in a graded ethanol series, and subjected to antigen retrieval with citrate buffer. After blocking with serum at room temperature, sections were incubated with primary antibodies at 4 °C overnight and incubated with secondary antibodies next day. Afterwards, the sections were briefly counterstained with diaminobenzidine (DAB) for the chromogenic reaction, and the nuclei were stained with hematoxylin. Eventually, slides were observed under an optical microscope (Nikon, Japan).

### Statistical analysis

All experiments were repeated at least three times and representative results are presented. Data are expressed as the mean ± SD. Statistical significance (*p < 0.05, **p < 0.01) was determined by two-tailed student *t*-test between two groups and One-way ANOVA analyses of variance among groups by SPSS 16.0.
